# The role of Guanxi in fostering adaptability and work engagement among educators in international educational institutions

**DOI:** 10.3389/fpsyg.2023.1336189

**Published:** 2024-01-04

**Authors:** Shuang Li, Shougang Yu

**Affiliations:** ^1^College of Teacher Education, Harbin University, Harbin, China; ^2^College of International Cooperation Education, Harbin Engineering University, Harbin, China

**Keywords:** adaptive performance, cross-cultural adaptation, Chinese culture, Confucian values, emotional bonds, Ganqing, Renqing, Xinren

## Abstract

The present study delves into the intricate relationships between individual adaptability, various dimensions of Guanxi—Ganqing, Renqing, and Xinren—and work engagement among educators working in international educational institutions across major cities in China. Guanxi refers to complex system of social networks and influential relationships that facilitate business and other dealings. Ganqing, Renqing, and Xinren represents distinct but interconnected dimensions. Ganqing refers to the emotional bonding or personal affection that is developed within a Guanxi relationship. Renqing can be understood as the norm of reciprocity or the social obligation to respond to another’s needs and to maintain the balance of give and take in relationships. Finally, Xinren: This dimension represents trust and credibility in Guanxi relationships. Employing online data collection via the Qualtrics platform, the study investigates the impact of individual adaptability on work engagement, particularly examining the mediating roles of different Guanxi dimensions. Utilizing Hayes’ Process Model 80 for mediation analysis, our findings demonstrate a statistically significant direct effect of individual adaptability on work engagement, thus supporting Hypothesis 1 (H1). The analysis reveals that this relationship is partially mediated by other variables within our model. Significantly, the study highlights the nuanced roles of the Guanxi dimensions of Ganqing, Renqing, and Xinren in this context. Both Ganqing and Renqing were found to amplify the effect of individual adaptability on work engagement, confirming Hypotheses 2a and 2b. However, while Xinren increased the effect size, it did not significantly mediate the relationship between individual adaptability and work engagement, leading to the rejection of Hypothesis 2c. Furthermore, our research provides new insights into the interplay between these Guanxi dimensions. Specifically, Ganqing and Renqing significantly influenced Xinren, which in turn impacted work engagement, thereby supporting Hypotheses 3a and 3b. This mediated chain model suggests a more complex interaction between these factors than previously understood. Our analysis also reveals the differential impacts of these Guanxi dimensions. Notably, Ganqing exhibited a greater influence on work engagement compared to Renqing and Xinren. This finding underscores the critical role of affective bonds in social ties and their importance in enhancing work engagement. These results, robust across statistical metrics including R, R-squared, MSE, F, and *p*-values, are detailed in our results section and illustrated in Figure 3. The study contributes to the understanding of how individual adaptability and various Guanxi dimensions interact to influence work engagement, offering valuable insights for both academic research and practical application in organizational settings. These findings are contextualized within Confucian values and the ongoing internationalization of education. The study thus advances the theoretical discourse while offering practical recommendations for educators and institutional policies. Limitations and future research directions are also elaborated.

## Introduction

The rapid globalization of education has led to transformative changes in international educational institutions, particularly in culturally diverse countries like China (Marginson, 2011; Yang, 2016). As these institutions broaden their curricula to offer programs such as the International Baccalaureate, American, and British systems, educators are increasingly navigating a complex blend of pedagogical philosophies and cultural norms (Knight, 2004; Deardorff and Jones, 2022). Within this evolving landscape, adaptability and work engagement have gained scholarly and practical interest (Martin et al., 2013; Bakker and Demerouti, 2017).

Adaptability, understood as the capability to effectively adjust to new situations and environments, has been emphasized as a crucial attribute for educators (Pulakos et al., 2000). Work engagement, on the other hand, pertains to the degree of enthusiasm, commitment, and vested interest an educator has toward their professional roles (Schaufeli and Bakker, 2004).

These constructs, though well-explored in general, lack an in-depth examination of their interplay within the unique cultural contexts of international education in China.

Herein lies the significance of “Guanxi,” a fundamental element of Chinese social and professional interactions, which potentially influences both adaptability and work engagement. “Guanxi” encompasses a complex network of social connections and relationships characterized by mutual trust, reciprocal favors, and emotional bonding (Chen and Chen, 2004; Liu et al., 2014). In the Chinese educational setting, this concept becomes particularly crucial as it shapes interactions and expectations among educators, students, and the wider school community. The dimensions of “Guanxi,” such as Ganqing (emotional bonds), Renqing (social norms), and Xinren (trust), are hypothesized to mediate the relationship between adaptability and work engagement, offering a nuanced understanding of how educators adjust and thrive in these culturally diverse environments. Ganqing is cultivated over time and is indicative of a genuine, deep emotional connection between individuals, going beyond mere superficial acquaintance. It is fostered through shared experiences, mutual understanding, and empathetic interactions. In the context of Guanxi, Ganqing is essential as it forms the foundation of trust and loyalty that underpins these relationships. Strong Ganqing in a relationship often leads to a higher level of trust and a stronger commitment to mutual support. Renqing embodies the idea that favors or assistance should be reciprocated, not necessarily immediately or in the same form, but in a manner that acknowledges and honors the relationship. Renqing is deeply ingrained in Chinese culture, guiding social interactions and the expectations individuals have in their relationships. It is a key driver in the maintenance and strengthening of Guanxi networks, as it dictates the behaviors and responses appropriate within these relationships. Xinren is earned over time and is crucial for the establishment and maintenance of strong, effective Guanxi networks. It is based on the consistent demonstration of integrity, reliability, and honesty. In a business context, Xinren is vital as it underpins successful and enduring partnerships. The presence of Xinren in a relationship implies a level of confidence that parties will act in good faith and uphold their commitments, thereby facilitating smoother and more effective interactions.

Each of these dimensions plays a vital role in the development, maintenance, and efficacy of Guanxi networks. Understanding these dimensions is essential for comprehending how Guanxi functions in Chinese social and business contexts and for effectively navigating these networks.

This study aims to explore this mediation effect using Hayes’ Process Model 80 on a sample of 603 educators from various international educational institutions in major Chinese cities (Hayes, 2022). By examining the role of “Guanxi” in the adaptability-engagement nexus, this research not only contributes to the theoretical understanding of these constructs but also provides practical insights for policy formulation and professional development in international education settings in China (Wang and Noe, 2010; Wilkins and Annabi, 2023). By exploring the relationships between individual adaptability, various Guanxi dimensions, and work engagement, this study seeks to offer actionable insights for educational institutions in crafting policies that foster a conducive work environment and improve educators’ wellbeing (Jie, 2023; Wang et al., 2023).

## Theoretical literature framework

### Educators in Chinese international educational institutions

Within the educational landscape, international educational institutions in China are distinguished by their curricula, often divergent from the Chinese national syllabus, and a diverse student demographic comprising both local and international students. These institutions are usually situated in major cities and accredited by international educational bodies, offering a variety of curricula like the International Baccalaureate, American, and British educational systems (Wilkins, 2020; Xu, 2023). Educators in these international settings assume roles far more complex than mere conduits of knowledge. They often serve as cultural mediators, mentors, and social network builders (Li and Eryong, 2022). Notably, their roles expand to include sensitivities toward varied educational philosophies and methodologies, making adaptability a highly valued skill (Day and Gu, 2010). Their function as the linchpin in cross-cultural communication is further complicated by the plurality of expectations stemming from students, parents, and the educational institution (Tschannen-Moran and Hoy, 2001).

This need for individual adaptability is further underscored by the diversity in cultures, educational philosophies, and methodologies that these institutions encapsulate (Hargreaves, 2000). Educators are often required to exhibit high levels of adaptability as they traverse different learning styles, cultural backdrops, and linguistic proficiencies (Ryan and Deci, 2000). Social relationships, vital for effective functioning within these academic settings, often find roots in the cultural construct of “Guanxi,” a form of social networking deeply ingrained in Chinese culture (Zhang and Zhang, 2006; Park and Chesla, 2007).

In summary, educators in international educational institutions in China navigate an intricate maze of cultural, pedagogical, and social demands. Their roles are not just multifaceted but also necessitate high adaptability and robust social networking skills (Wang et al., 2023). These characteristics are heightened by the inherently diverse nature of such academic settings, making the exploration of adaptability and social relationships in relation to work engagement an imperative area for academic scrutiny.

### Individual adaptability in educational settings

Adaptability is frequently conceptualized as the ability to modify one’s cognitive processes, behavioral responses, and emotional states in reaction to shifting conditions. This attribute takes on elevated importance in multifaceted and dynamic organizational contexts, such as international educational institutions. According to Pulakos et al. (2000), individual adaptability constitutes a distinct dimension of job performance, diverging from both task and contextual performance. Subsequent research has supported this conceptual framework, reinforcing its standing as an individual yet integral component of overall job efficacy (Pulakos et al., 2002).

In operational terms, adaptability entails a synthesis of multiple cognitive and non-cognitive attributes. These include meta-cognitive skills, problem-solving capabilities, decision-making acumen, self-awareness, resilience, tolerance for ambiguity, openness to experience, motivation for achievement, practical experience, and domain-specific knowledge (White et al., 2005). In the context of international educational institutions, which are marked by a mélange of cultural backgrounds, pedagogical philosophies, and teaching methodologies, the need for adaptability is acutely felt. Educators in these settings confront the necessity to be adaptable both in their instructional techniques and in their social interactions to navigate this complex landscape effectively (Tschannen-Moran and Hoy, 2001; Rai et al., 2023).

Research has consistently underscored the significance of individual adaptability in predicting favorable workplace outcomes, such as enhanced work engagement (Deci et al., 2017). This is particularly evident in the dynamic context of international educational institutions, where the adaptability of educators extends its influence beyond personal job satisfaction. It critically shapes the overall effectiveness and efficiency of these institutions (Day and Gu, 2010).

In the current global milieu, marked by unprecedented challenges such as the COVID-19 pandemic, the importance of adaptability in educational settings has been profoundly amplified. The pandemic has not only disrupted traditional teaching methodologies but also imposed novel psychological and logistical challenges on educators. This disruption necessitates a reevaluation of adaptability, especially in the way it influences educators’ ability to maintain engagement and productivity in the face of rapid and unforeseen changes.

Drawing on the theoretical framework provided by Pulakos et al. (2002), it becomes essential to explore how adaptability manifests in this new context. The COVID-19 pandemic has radically altered the landscape of international education, presenting multifaceted challenges that educators must navigate. These include transitioning to online teaching platforms, modifying curriculum delivery to accommodate remote learning, and managing the psychological impacts of prolonged uncertainty and isolation. Consequently, the adaptability of educators now plays a pivotal role in not only sustaining their job satisfaction but also in ensuring the continued effectiveness of educational institutions during and beyond the pandemic era.

By examining adaptability through this lens, we can gain a deeper understanding of the complexities and opportunities that educators in international educational settings face. This approach enables a more nuanced appreciation of how adaptability serves as a crucial determinant of educational resilience and success in a rapidly evolving global landscape shaped by COVID-19.

### Educators’ work engagement under the job demands-resource model

Work engagement, broadly defined, refers to a positive, fulfilling, work-related state of mind characterized by vigor, dedication, and absorption (Mazzetti et al., 2023). Within educational settings, work engagement becomes particularly crucial for educators, as it not only impacts their wellbeing but also significantly influences the learning outcomes for students. Educators who are highly engaged are often more committed, enthusiastic, and effective in their roles, thereby contributing to the overall quality of education provided (Schaufeli and Bakker, 2004; Bakker and Demerouti, 2008, 2017).

The Job Demands-Resource Model (JD-R Model) offers a comprehensive framework for understanding work engagement by categorizing occupational factors into two primary categories: job demands and job resources. Job demands refer to the physical, psychological, social, or organizational aspects of the job that require sustained physical or mental effort, potentially leading to strain. Conversely, job resources refer to those physical, psychological, social, or organizational aspects that help in achieving work goals, reduce job demands, and stimulate personal growth (Bakker and Demerouti, 2007). Within the educational context, job demands might encompass high workload, emotional demands, and behavior management, while job resources could include professional development opportunities, supportive colleagues, and constructive feedback.

Given the increasing challenges in contemporary workplaces, such as growing cultural diversity, technological changes, and a recent surge in remote work prompted by global events, the relevance of work engagement, as described by the JD-R model, is increasingly apparent. High levels of work engagement are correlated with numerous positive outcomes for both workers and organizations. For workers, it contributes to wellbeing, job satisfaction, and reduced levels of burnout (Hakanen et al., 2006). For organizations, engaged employees contribute to higher levels of productivity, lower absenteeism, and increased customer satisfaction (Xanthopoulou et al., 2009).

The study conducted by Park et al. (2020) provides critical insights into the relationship between adaptive performance and work engagement, particularly within the context of human resources professionals in South Korea. This research highlights a significant link where adaptive performance not only emerges as a consequence of work engagement but also as a facilitator of it. Central to this study is the concept that adaptive performance is intricately connected to how deeply employees are engaged with their work. Park et al. (2020) utilized structural equation modeling to analyze data from 250 professionals, and their findings indicate that higher levels of adaptive performance are associated with increased work engagement. This relationship is partly mediated by the extent of job crafting, which refers to the proactive alterations employees make in their job roles.

The study reveals that when employees exhibit adaptability, it positively influences their level of engagement with work tasks. This increased engagement, in turn, fuels further adaptive performance, creating a synergistic cycle. In essence, adaptability enhances an employee’s ability to engage with their work, and this heightened engagement feeds back into their capacity for adaptability.

Furthermore, Park et al. (2020) demonstrate that this linkage between adaptive performance and work engagement is part of a larger dynamic involving organizational support. Organizational support facilitates job crafting, which then leads to enhanced work engagement and subsequently improves adaptive performance. This indicates that adaptive performance is both a precursor and a product of work engagement, suggesting a complex, bidirectional relationship.

In order to achieve work engagement, personal adaptability seems necessary. As pointed out by Marlow (2016), the fit between Adaptability and the requirements of the job was shown to successfully predict engagement when taken as an overall fit as well as within the dimensions of openness to criticism and flexibility of opinion. Moreover, among the 331 participants in the study, the link between conscientiousness and engagement was shown to be partially mediated by Adaptability.

In conclusion, the findings from previous research provide compelling evidence for the interconnectedness of adaptive performance and work engagement. This study underscores the pivotal role of adaptive performance in not only being an outcome of high work engagement but also in promoting and sustaining it within organizational settings.

### Guanxi as social and task resources for educators

The term “Guanxi,” rooted in Chinese society, refers to an intricate network of social relationships that serve both personal and professional goals. In academic literature, Guanxi is generally understood to encompass three dimensions: Ganqing, Renqing, and Xinren. Ganqing refers to the emotional attachment between individuals in a Guanxi network, often akin to loyalty or bonding. Renqing involves the concept of social obligation and reciprocity, such as doing favors or offering gifts. Xinren refers to the dimension of trust and credibility within the Guanxi network (Hwang, 1987; Yang, 2016).

In Chinese society, Guanxi is not merely a cultural idiosyncrasy but serves as a functional mechanism that permeates various facets of life, including workplace relationships and behaviors (Bian and Ang, 1997). This is particularly relevant in international educational institutions in China, where educators often work in a multicultural environment. Here, Guanxi serves dual roles as both social and task resources. As a social resource, Guanxi aids in integrating diverse cultures and promotes an understanding of various educational philosophies. This assists in mitigating the strain of emotional labor often involved in teaching roles (Hobfoll, 2001). As a task resource, Guanxi can help educators in career progression and networking, thereby providing them with the tools necessary for teaching effectiveness and educational leadership (Wilkins and Annabi, 2023).

Recent research further augments this understanding by integrating Guanxi into occupational stress models. For instance, Hu et al. (2016) extended the Job Demands-Resources model by incorporating Guanxi as a form of social exchange. This extension underscores the capacity of Guanxi to serve as a resource that can enhance work engagement and reduce job strain.

Given the increasingly global nature of workplaces and the confluence of varied cultural philosophies in international educational settings, the utility of Guanxi has become more salient than ever. Understanding how Guanxi serves as a social and task resource can provide valuable insights into improving educators’ work engagement and overall workplace wellbeing (Hobfoll, 2001; Halbesleben, 2010; Mazzetti et al., 2023).

### The mediating role of Xinren between other Guanxi’s dimensions and outcomes

The concept of “Xinren,” often translated as trust or credibility, serves as an indispensable element in the intricate tapestry of Guanxi in Chinese society. Xinren is so integral to Chinese culture that it not only reflects personal relationships but also shapes business interactions and workplace dynamics (Chen and Chen, 2004; Luo et al., 2012). While Guanxi is often dissected into various dimensions, including Ganqing, Renqing, and Xinren, the role of Xinren frequently emerges as both an outcome and a mediator.

The achievement of Xinren can be considered an ultimate goal of the Guanxi mechanism, emerging from the intricate interactions of its other dimensions. Ganqing and Renqing create the foundation upon which Xinren is built, essentially serving as social investments that yield the return of trust (Hwang, 1987; Yang, 2016). Once achieved, Xinren brings about a multiplier effect, transforming a network of social relationships into a genuinely potent instrument for personal and professional advancement (Tsui and Farh, 1997; Chen and Bedford, 2022).

The interconnectedness of Ganqing, Renqing, and Xinren within the Guanxi framework operates in a hierarchical and symbiotic manner. Ganqing and Renqing lay the groundwork for Xinren by establishing emotional bonds and reciprocal obligations, which are essential for cultivating a deep-seated sense of trust and credibility. Ganqing, characterized by emotional attachment and personal bonding, creates an atmosphere of mutual understanding and empathy. This emotional connection is critical in fostering an environment where individuals feel valued and understood. Renqing, which revolves around social obligation and reciprocity, further strengthens these bonds by encouraging a culture of mutual support and collaboration. These acts of giving and receiving help, guidance, or support are not just transactional exchanges; they are profound investments in the relationship, deepening the sense of commitment and reliability among individuals.

Xinren, the culmination of these interactions, embodies the trust and credibility that emerge from the foundations laid by Ganqing and Renqing. It is within this context that Xinren becomes pivotal in uncertain environments, serving as a critical component for adaptability. Trust, as encapsulated by Xinren, facilitates open communication, reduces the fear of vulnerability, and promotes a willingness to embrace change. In settings characterized by uncertainty, such as rapidly evolving educational or professional landscapes, trust becomes a cornerstone for making decisions and taking actions. When individuals trust their colleagues, leaders, or the organizational ethos, they are more inclined to adapt to new situations, embrace innovative approaches, and take calculated risks. This trust-based adaptability is not just about coping with change; it is about thriving in it.

Furthermore, Xinren’s role in adaptability extends beyond individual interactions. It permeates the entire organizational or institutional culture, creating an environment where adaptability is not just encouraged but ingrained. In such settings, trust-based relationships enable individuals to share knowledge freely, collaborate effectively, and approach challenges with a solutions-oriented mindset. This collective adaptability, rooted in a culture of trust, is particularly crucial in educational settings, where the ability to adapt to diverse student needs, pedagogical advancements, and cultural nuances can significantly enhance educational outcomes. Thus, Xinren, nurtured through the emotional connections of Ganqing and the reciprocal bonds of Renqing, emerges as a fundamental element in fostering an adaptive, resilient, and dynamic environment.

In the context of educational settings, particularly among educators in international institutions, Xinren holds additional significance. It acts as a mediating variable that significantly impacts the relationship between adaptability and work engagement (Guo et al., 2022). When educators successfully cultivate Xinren through effective Guanxi, they find themselves better equipped to adapt to diverse work environments. This adaptability, in turn, heightens their engagement levels, thereby impacting the quality of education and the wellbeing of both educators and students (Chen and Tjosvold, 2012).

Theoretically, Xinren serves as both an outcome and a mediator, transforming a Guanxi-based social network into a functional system that impacts multiple layers of workplace behavior and emotional wellbeing. Its dual role merits closer scrutiny, especially in international educational contexts that are subject to the influence of various cultural and organizational elements.

Based on the above reviewed literature, in the present study the following hypotheses are proposed:

*H1*: Individual Adaptability predicted Work Engagement.

*H2*: Guanxi (Ganqing H2a, Renqing H2b, and Xinren H2c) mediates the relationships between Adaptability and Work Engagement.

*H3*: Xinren mediates the relationships between both Individual Adaptability and Ganqing (H3a), and Renqing (H3b), on the one side, and Work Engagement, on the other.

**Figure 1:**
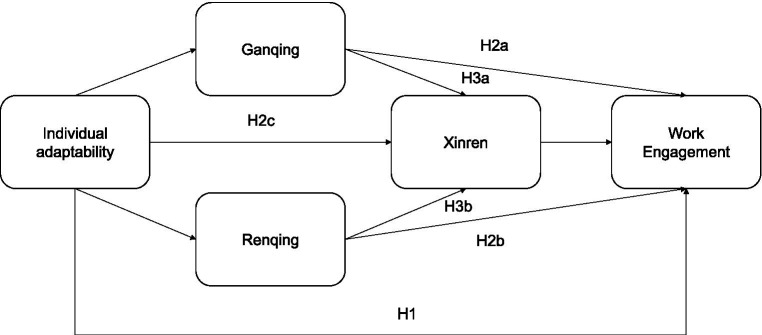
Hypothesized Model 80 for the present study.

The global model for this study is displayed in Figure 1.

## Methods

### Participants and procedure

The sample of the current study was composed of 603 educators from international educational institutions situated in major Chinese cities, offering diverse curricula such as International Baccalaureate (IB), American, and British programs. The participant pool was varied, encompassing different departments, seniority levels, subjects, and roles within the academic hierarchy. Mean age was 57.19 (*SD* = 7.18), 50.4% were males and, regarding the academic hierarchy, junior roles (Teaching Assistants or Research Assistants) were 69%, Lecturers were 16.9%, Associate Professors were 11.8%, and Full Professors were only 2.2%. The percentage of males within the academic hierarchy levels ranges between 73.5% among Lecturers and 46.2% among the junior roles. The underrepresentation of females in senior academic positions is not unique to China and is a phenomenon observed in many countries.

Data collection was conducted online through the Qualtrics platform, a method that not only facilitated participant access but also streamlined data management for the research team.

Prior to distributing the survey, ethical approval was obtained from the Ethical Committee of the Harbin University has been obtained. An informed consent form was presented to potential participants at the outset of the survey, detailing the research aims, ethical considerations, including the confidentiality and voluntary nature of participation, and anticipated time commitment. The initial number of potential participants contacted via email invitation was 1,530 educators, form them, a total of 897 responses were received. Emails containing uniquely generated survey links were sent to educators to safeguard confidentiality, and this email was sent to the 897 potential participants. The email reiterated the study’s purpose, time commitment, and ethical considerations, such as the assurance of anonymity and voluntary participation. Periodic email reminders were dispatched during the two-month data collection period. The total number of received responses was 709 (79% response rate), but some responses were excluded due that the percentage of the survey completion was not 100%.

### Instruments

#### Individual adaptability

The instrument employed for measuring Individual adaptability was based in Grim’s study (2010), known as the *Supervisor Ratings of Adaptive Performance*. In this evaluative measure, supervisors assessed their subordinates’ adaptive performance capabilities using a 5-point Likert scale. The instrument consists of 15 behavior-based statements, each aligned with one of the five key dimensions of adaptive performance: (1) Handling work stress; (2) Creative problem-solving; (3) Navigating uncertain and unpredictable work conditions; (4) Acquiring new work tasks, technologies, and procedures; and (5) Demonstrating interpersonal adaptability. Each of these dimensions is represented by three specific items, the wording for which was adapted from the definitions of adaptive performance dimensions originally formulated by Pulakos et al. (2000). This methodology closely mirrors that employed by Pulakos and colleagues, where they used a similar instrument to gauge adaptive performance ratings from military supervisors (Pulakos et al., 2002). In the context of the original study, the instrument demonstrated remarkable internal consistency, as evidenced by a Cronbach’s alpha value of 0.98 across both samples, and in the present study alpha was 0.839, while McDonald’s *ω* = 0.838. The specific layout of this supervisor-based evaluation tool can be found in Table 1. As in the original study (Grim, 2010), a global assessment of Individual adaptability has been obtained.

**Table 1:** Individual adaptive scale: factor loadings and items content.

Items	Factor 1 (Creative problem-solving)	Factor 2 (Acquiring new work tasks, technologies, and procedures)	Factor 3 (Demonstrating interpersonal adaptability)	Factor 4 (Handling work stress)	Factor 5 (Navigating uncertain and unpredictable work conditions)
Generates new, innovative ideas to solve complex problems	0.867				
Develops innovative methods of obtaining resources to get the job done	0.646				
Turns problems upside-down and inside-out to find fresh, new approaches	0.626				
Demonstrates enthusiasm for learning new skills and technology		0.738			
Quickly and proficiently learns new ways to perform previously unlearned tasks		0.678			
Volunteers to attend training that will prepare self for new skills needed at work		0.579			
Flexible and open-minded when dealing with others			0.562		
Works well and develops effective relationships with people with different personalities			0.776		
Demonstrates keen insight of others’ behavior and adjusts own behavior to be able to work more effectively with them			0.715		
Manages frustration well by working toward a solution, rather than blaming others				0.507	
Remains composed and cool when faced with difficult circumstances				0.621	
Does not overact to unexpected situations				0.462	
Refuses to be frozen or paralyzed by uncertainty					0.571
Takes effective action, even when the situation is not clear					0.556
Readily and easily changes gears in response to unexpected changes					0.254

#### Work engagement

In the current study, work engagement was assessed using the Utrecht Work Engagement Scale-3 (UWES-3). Developed by Schaufeli and Bakker (2004), this abbreviated instrument is a streamlined version of the original Utrecht Work Engagement Scale. It comprises three items, each corresponding to one of the three key dimensions of work engagement: Vigor, Dedication, and Absorption. Participants are asked to rate their agreement with each item on a 7-point Likert scale, ranging from 0 (*Never*) to 6 (*Always*). The items are designed to measure the extent to which individuals are energetic and mentally resilient while working (Vigor), derive a sense of significance and enthusiasm from their work (Dedication), and are fully engrossed in their work activities (Absorption).

The UWES-3 has been validated across diverse cultural and occupational settings, demonstrating both good reliability and construct validity. In the present research, the internal consistency reliability of the UWES-3 was found to be satisfactory, with a Cronbach’s alpha of *α* = 0.91. Previous studies have also substantiated the predictive validity of the UWES-3, confirming its capacity to predict a range of positive occupational outcomes, including job performance and organizational commitment (Mazzetti et al., 2023). The three items of the questionnaire are: “At my work, I feel bursting with energy” (vigor); “I am enthusiastic about my job” (dedication); and “I am immersed in my work” (absorption). Despite that a plethora of studies considers Work Engagement as multidimensional, some previous study (Hallberg and Schaufeli, 2006) indicated that the inter-correlations among the uni-dimensional representation of the construct evidenced an equivalent fit to data compared to the original three-factor solution. In a similar vein, there were high and significant correlations between the three, theoretically separate, dimensions of work engagement (Kulikowski, 2017). In data sets from 10 different countries, median correlations between 3 various dimensions were higher than 0.9. In addition, some researchers have drawn attention to a lack of validity for the three-factor UWES (Viljevac et al., 2012). Hence, work engagement was conceptualized as a uni-dimensional construct for statistical reasons in later studies as Hallberg et al. (2007), and we followed this procedure.

#### Guanxi

In the current investigation, the construct of Guanxi was assessed through the Guanxi Quality Scale (GQS), a psychometrically robust 15-item instrument specifically designed to evaluate the quality of Guanxi relationships within the workplace (Chen and Bedford, 2022). The scale encompasses three dimensions: Ganqing, Renqing, and Xinren, each represented by five items. Participants are required to express their level of agreement with these items using a Likert scale. The Ganqing dimension captures emotional connection and loyalty, featuring a Cronbach’s alpha of 0.90 in the study on their psychometric properties (Chen and Bedford, 2022), indicative of excellent internal consistency. An item example is “Our interaction is not limited to the scope of work.” The Renqing dimension concerns social obligations and reciprocity, and it too shows commendable internal consistency, with a Cronbach’s alpha of 0.90 (Chen and Bedford, 2022). Example is: “If he/she has helped me before, I will help him/her in turn.” Finally, Xinren, the second dimension, focuses on trustworthiness and reliability, and it also demonstrates high internal consistency with a Cronbach’s alpha of 0.90 (Chen and Bedford, 2022). An item of example is: “I trust him/her.” The construct validity of the GQS is further corroborated when compared to similar scales. For instance, Yen et al. (2011) devised another instrument that similarly posits three dimensions of Guanxi: Ganqing (affect), Renqing (reciprocal favor), and Xinren (trust). The parallel between these dimensions and those of the GQS suggests a convergence of scholarly perspectives on the facets of Guanxi, thereby enhancing the validity of the GQS in capturing this culturally nuanced construct. In the present study, global reliability has been adequate, both for Cronbach’s Alpha *α* = 0.891 and McDonald’s *ω* = 0.869. For the Guanxi’s dimensions, Ganqing (Cronbach’s Alpha *α* = 0.844 and McDonald’s *ω* = 0.847), Renqing (Cronbach’s Alpha *α* = 0.808 and McDonald’s *ω* = 0.814), and Xinren (Cronbach’s Alpha *α* = 0.898 and McDonald’s *ω* = 0.870).

### Data analyses

Data were analyzed using SPSS version 24, JASP version 0.18.1 (JASP Team, 2023). JASP has been used for the Exploratory and Confirmatory Factor Analyses. PROCESS 4.2 macro (Hayes, 2022) was utilized to examine the chain mediation in our research. This macro is a versatile computational tool designed for the analysis of mediating, moderating, and conditional process models in statistical research. The chain mediation model, particularly Model 80, is adept at testing complex relationships involving multiple mediators in a sequential arrangement. Our analysis began with establishing the basic relationships between the variables under consideration—Individual Adaptability, Work Engagement, and the Guanxi dimensions (Ganqing, Renqing, and Xinren)—through Pearson’s correlation. This step was critical to determine the strength and direction of the linear relationships among these variables.

Subsequently, we employed Model 80 of the PROCESS macro to conduct a chain mediational analysis. This model is specifically designed to test hypotheses involving multiple mediators that are linked sequentially. In our case, the model tested the hypothesis where Xinren’s mediating effect on the relationship between Individual Adaptability and Work Engagement is preceded by the mediation of Ganqing and Renqing. Essentially, it examined whether the impact of Individual Adaptability on Work Engagement is transmitted through Ganqing and Renqing, and then through Xinren in a sequential manner. One of the key aspects of this analysis was the use of bootstrapping, a resampling technique, to estimate the indirect effects of the mediators. We employed 5,000 bootstrap re-samples to ensure the robustness of our estimates. This non-parametric approach allows for the approximation of the sampling distribution of the indirect effects, thereby providing more accurate confidence intervals.

The significance of the chain mediation hypothesis was determined based on the 95% bias-corrected confidence intervals derived from the bootstrapping procedure. If the confidence interval for the indirect effect does not include zero, it suggests that the mediating effect is statistically significant at *p* < 0.05. This means that there is a less than 5% probability that the observed effect is due to chance, thereby lending credibility to the mediating role of the Guanxi dimensions in the relationship between Individual Adaptability and Work Engagement.

In summary, the use of Hayes Model 80 within the PROCESS 4.2 macro facilitated a comprehensive and statistically rigorous examination of the hypothesized chain mediation effect in our study. This approach not only adhered to the principles of robust statistical analysis but also allowed for a nuanced understanding of the complex interplay between the variables under investigation.

## Results

### Confirmatory factor analyses

Individual Adaptability Scale has been submitted to Exploratory Factor Analysis (EFA), given that the original studies affirmed that five dimension of the Pulakos’ taxonomy was represented by three items, but the results were not published. The EFA was conducted by JASP using the maximum likelihood method for factoring and the Varimax rotation. The cumulative explained variance for the five factors solution was 52.3%. Five factors emerged with three items, as Table 1 showed.

**Figure 2:**
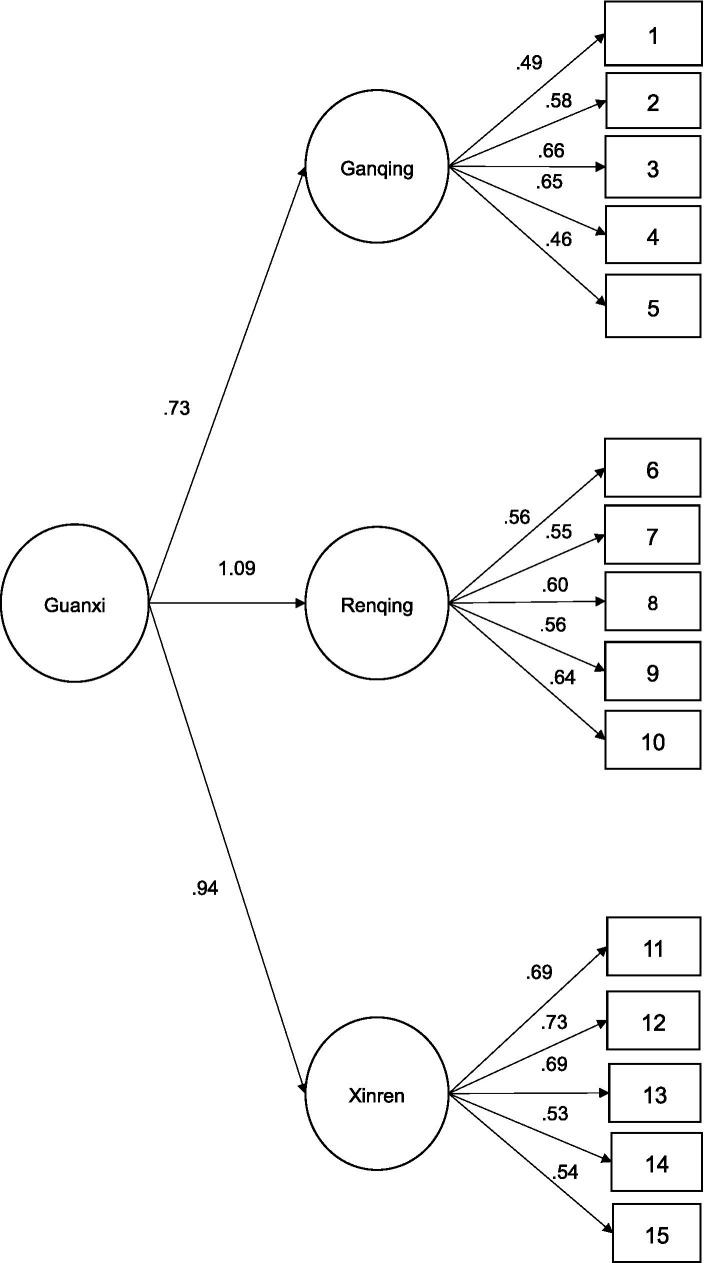
Confirmatory factorial analysis of GQS.

For the GQS scale, as Confirmatory Factor Analyses (CFA) was provided by the original study (Chen and Bedford, 2022), also CFA was conducted using JASP. Previously, the Kaiser-Meyer-Olkin (KMO = 0.881), and the Bartlett’s sphericity test (Chi Square = 5211.87, df = 105, *p* < 0.001) have been tested and both of them showed adequate values. The model with only one factor showed inadequate fit indices (Chi-Square = 2228.56, df = 90, *p* = 0.000, RMSEA = 0.199, SRMR = 0.147). The model with three related factors showed better fit (Chi Square = 510.605, df = 87, *p* < 0.001). The Average Extracted variance was Guanxi dimensions, Ganqing (0.532), Renqing (0.475), and Xinren (0.629). The factorial loadings showed adequate values for all the factors, as Figure 2 showed. Following the proposal of the original study (Chen and Bedford, 2022), a second order factor model has been tested. The three dimensions showed adequate factor loadings, being statistically significant, as can be seen in Table 2 and Figure 2.

**Table 2:** Second-order factor loadings.

						95% confidence interval	
Second-order Factor	First-order factor	Estimate	Std. Error	*z*-value	*p*	Lower	Upper
Guanxi	Ganqing	0.734	0.082	8.995	< 0.001	0.574	0.894
	Renqing	1.086	0.146	7.446	< 0.001	0.800	1.372
	Xinren	0.944	0.112	8.424	< 0.001	0.724	1.164

### Mediated chain model

**Figure 3:**
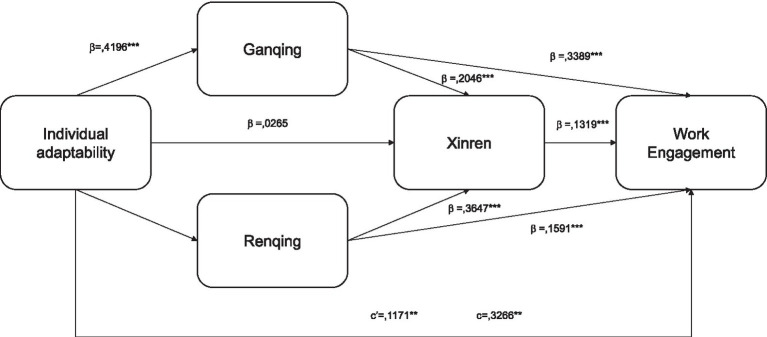
Standardized estimates for the Model 80. **p < 0.01; ***p < 0.001. β, standardized estimates; c’, direct standardized estimate from X to Y; c, total standardized estimate from X to Y.

We conducted a mediation analysis using Hayes’ Process Model 80 to evaluate the chain mediating effects within our model. The statistical metrics, including *R* = 0.3266; R-sq = 0.1067; MSE = 0.5677; *F* = 71.7577; df1 = 1; df2 = 601; *p* = 0.0000, affirmed the robustness of the baseline model. Figure 3 indicate that both the model and its corresponding results are reliable for interpretation. The total effect of X (Individual Adaptability) on Y (Work Engagement) was statistically significant (Effect = 0.4715; se = 0.0557; *t* = 8.4710; *p* = 0.000; 95% LLCI = 0.3622; ULCI = 0.5808; c completely standardized effect = 0.3266).

Upon closer examination of the data, it was observed that the direct effect of Individual Adaptability on Work Engagement was significant (Effect = 0.1691; se = 0.0543; *t* = 3.1169; *p* = 0.0019; 95% LLCI = 0.0626; ULCI = 0.2.757; c’ completely standardized effect = 0.1171), thus supporting Hypothesis 1 (H1). This suggests that other variables in the model partially mediate the relationship between Individual Adaptability and Work Engagement.

The analysis then progressed to scrutinize each mediating chain of influence. Specifically, both Ganqing and Renqing amplified the effect of Individual Adaptability on Work Engagement, supporting Hypothesis 2 (H2a, and H2b). However, Xinren, although increasing the effect size, did not significantly mediate between Individual Adaptability and Work Engagement, leading to the rejection of Hypothesis 2 (H2c). On the other hand, Ganqing was found to significantly impact on Xinren, which subsequently influenced Work Engagement, thereby supporting Hypothesis 3 (H3a). Renqing was also found to significantly impact Xinren, that later mediate the relationship between Individual Adaptability and Work Engagement, supporting Hypothesis 3 (H3b). These findings are elaborated in Table 3.

**Table 3:** Standardized indirect effects.

Standardized indirect effects	Effect	BootSE	Boot LLCI	BootULCI
Total	0.3024	0.0410	0.2255	0.3857
Individual adaptability-> Ganqing- > work engagement	0.2053	0.0348	0.1419	0.2776
Individual adaptability- > Renqing- > work engagement	0.0595	0.0201	0.0248	0.1040
Individual adaptability- > Xinren- > work engagement	0.0032	0.0092	−0.0133	0.0241
Individual adaptability- > Ganqing- > Xinren- > work engagement	0.0163	0.0066	0.0052	0.0304
Individual adaptability- > Renqing- > Xinren- > work engagement	0.0180	0.0065	0.0062	0.0317

Moreover, the study revealed nuanced roles for different variables in mediating the effect of Individual Adaptability on workers’ Engagement. Specifically, increased Work Engagement by enhancing Xinren, and also serve as a standalone mediator. In the same vein, Renqing enhances Xinren, as well as serves as mediator on the relationships between Individual adaptability and Work Engagement. In contrast, Xinren not directly mediated the relationship between Adaptability and Work Engagement but augmented the effect on Work Engagement. Importantly, Ganqing exhibited a greater impact on Work Engagement compared to other Guanxi’s dimensions, highlighting the role of affective bond on social ties. These observations are further illustrated in Figure 3.

## Discussion

The present study contributes to a nuanced understanding of the interplay between individual adaptability, dimensions of Guanxi (Ganqing, Renqing, and Xinren), and work engagement among educators in international educational institutions in China. The robustness of the baseline model was statistically confirmed, providing a reliable foundation for interpreting the study’s findings.

The significant direct effect of individual adaptability on work engagement aligns well with Confucian values, particularly the principle of “Yi” or righteousness. According to Confucianism, adaptability is not merely a function of survival but is tied to moral integrity and societal harmony (Bell, 2010). This ethical dimension resonates with the study’s finding that individual adaptability directly influences work engagement, thereby supporting Hypothesis 1 (H1).

The study further reveals the mediating role of Ganqing and Renqing in the relationship between individual adaptability and work engagement, confirming Hypotheses 2a and 2b. The mediating role of Guanxi dimensions—Ganqing and Renqing—between individual adaptability and work engagement is particularly illuminating. These dimensions resonate with the Confucian principle of “Ren,” which emphasizes compassion and humaneness in interpersonal relationships. Guanxi, in this context, is not just a network of relationships but a reflection of deep-seated cultural values that influence behavior and attitudes in professional settings. In international educational institutions in China, where cultural dynamics are complex and diverse, these dimensions of Guanxi become even more critical. Ganqing and Renqing, therefore, mediate the relationship between adaptability and engagement by fostering an environment where educators feel emotionally connected and morally obliged to each other and the institution. This enhances their engagement and commitment to their roles, as they navigate the challenges of a multicultural educational environment (Tu, 1985). Interestingly, Xinren did not significantly mediate between individual adaptability and work engagement, refuting Hypothesis 2c. This can be interpreted through the lens of Confucian skepticism toward blind trust. Confucianism favors a system of mutual obligations and moral integrity over mere trust without basis. In the context of international educational settings, this suggests that while adaptability is essential, it needs to be grounded in moral integrity and reciprocal obligations for it to effectively enhance work engagement (Tan and Snell, 2002). In light of the current trends of internationalization, the study’s findings gain additional relevance. Educators in international settings act as cultural intermediaries. Their ability to adapt and engage effectively is pivotal in blending diverse educational philosophies and practices. Our research suggests that leveraging the indigenous cultural construct of Guanxi could significantly enhance this adaptability and engagement, especially in contexts where maintaining a balance between global pedagogical approaches and local cultural values is crucial.

Our research consistently highlights the relationship between individual adaptability and work engagement. However, it is noteworthy that previous studies have predominantly examined this relationship in reverse. Notably, previous studies (Kaya and Karatepe, 2020; Buttigieg et al., 2023) explored how engagement acts as a precursor to adaptive performance. Additionally, Van den Heuvel et al. (2020) delved into the role of engagement in facilitating employees’ adaptation to organizational changes. These findings align intuitively with the understanding that for adaptive performance to manifest, individuals must prioritize effectively and navigate new challenges.

This body of research suggests that employees exhibiting high levels of vigor—characterized by energy and mental resilience—are better equipped for adaptability. Similarly, those who demonstrate dedication in their roles are more likely to adapt effectively. This implies that the qualities of vigor and dedication, integral components of work engagement, are crucial for optimal adaptive performance. Essentially, employees who are energized, mentally robust, and dedicated are positioned to exhibit superior adaptability in the face of evolving work demands and organizational changes.

In the era of increasing internationalization, the study assumes added relevance. Educators in international educational institutions in China serve as cultural bridges, and their adaptability and engagement are key to the successful fusion of diverse educational philosophies and practices. The study’s findings suggest that tapping into the native cultural construct of Guanxi could be instrumental in enhancing this adaptability and engagement. This is particularly crucial as international institutions strive to incorporate a global pedagogical outlook without diluting local cultural ethos (Knight, 2004). In summary, the study not only advances our empirical understanding of the relationship between individual adaptability, Guanxi, and work engagement but also contextualizes these findings within the broader framework of Confucian values and the current wave of internationalization (Lv et al., 2022; Rai et al., 2023). It thus offers valuable insights for both academics and practitioners interested in optimizing work engagement in culturally diverse educational settings.

### Limitations and suggestions for future research

While the study offers valuable insights into the relationship between individual adaptability, Guanxi, and work engagement in international educational settings in China, it is important to acknowledge its limitations. One notable constraint pertains to the methodology employed. Utilizing Hayes’ Process Model 80 for mediation analysis provides a rigorous statistical foundation, but it is not without its shortcomings, such as the assumption of linear relationships among variables. Further studies may benefit from employing alternative models or methodologies to examine these relationships from different perspectives (Hayes, 2022).

The study’s focus on educators in international educational institutions in China could also be seen as a limitation in terms of generalizability. While the findings are highly relevant to this specific context, they may not be directly applicable to educators in other types of institutions or in different cultural environments. Future research could therefore extend the study to other educational settings, either within China or internationally, to examine the universality of the findings (Xu and Connelly, 2022). Related to the instruments’ psychometric properties, we acknowledge that the fifteenth item of the scale measuring adaptability only reached a limited factor loading. Despite this, the decision to retain it has been based both on the relevance of having at least three observable indicators for each subtle dimension, an on the desire of maintaining the fidelity to the original study for offering comparable findings. Future research should explore the content of the item when translated to Chinese as well as test its psychometric properties. Another limitation could be the absence of a longitudinal design. The cross-sectional nature of the study offers a snapshot of the relationships among the variables but does not capture how these relationships evolve over time. Given the importance of Guanxi and its different dimensions in Chinese society, a longitudinal approach could provide more nuanced insights into how these variables interact over extended periods (Li and Eryong, 2022).

In this study, a notable gender imbalance was observed in the composition of the sample, with the percentage of males in academic positions ranging from 73.5% among Lecturers to 46.2% in junior roles. This disparity presents a limitation in our research, as it may influence the generalizability and interpretation of our findings, particularly concerning the dynamics of Guanxi within the academic context.

The predominance of male participants in our sample is reflective of a broader, global trend in academia, where women are underrepresented, especially in senior positions. This pattern is not unique to the Chinese academic system but is a phenomenon observed worldwide. The implications of such a gender imbalance are multifaceted. Primarily, it could lead to a skewed understanding of how Guanxi operates within academic settings, as gender can play a significant role in the formation and utility of these social networks. The perspectives and experiences of female academics in developing and leveraging Guanxi might differ substantially from their male counterparts, a nuance that our study could not fully explore due to the sample composition.

Moreover, the gender disparity in our sample limits the exploration of how gender dynamics influence the efficacy and nature of Guanxi in professional advancement and job satisfaction within academia. This gap highlights a need for further research that specifically focuses on the experiences of women in academic settings. Future studies should aim to understand how gender impacts the formation and benefits of Guanxi, potentially offering insights into strategies to enhance career progression and workplace satisfaction for female academics.

In conclusion, while our study provides valuable insights into the role of Guanxi in academia, the gender imbalance in our sample restricts the breadth of our conclusions. We recommend that subsequent research in this area adopt a more balanced gender approach to fully capture the diverse experiences and implications of Guanxi in academic settings.

The study’s findings regarding the non-significant mediating role of Xinren between individual adaptability and work engagement open up avenues for future research. This aspect warrants a more in-depth exploration, possibly through qualitative methodologies, to understand the underlying factors that may explain this unexpected outcome. Given that Xinren is rooted in Confucian values, which emphasize the importance of trust and integrity in relationships, a qualitative investigation could provide richer contextual understanding (Li et al., 2023). Lastly, the study has implications for the internationalization of education. As educational institutions globally become more diverse, understanding the dynamics of adaptability and engagement among educators becomes increasingly critical.

In summary, while the study offers compelling insights into the variables affecting work engagement among educators in international educational institutions in China, it also points to several avenues for future research. These include employing alternative methodologies, expanding the study’s scope to other educational and cultural contexts, adopting a longitudinal design, and delving deeper into the role of Xinren and the implications of internationalization.

### Suggestions for application in the educational context

The findings of this study have significant practical implications for both educators and the institutions they serve in. These suggestions are particularly relevant for international educational settings in China, where the dynamics of adaptability, Guanxi, and work engagement are of critical importance.

Educators are encouraged to actively foster their adaptability skills, given the study’s finding that individual adaptability significantly influences work engagement. This could entail continuous professional development courses or workshops focused on adaptability in cross-cultural contexts. Moreover, educators should consider actively engaging in the social fabric of their institutions to nurture relationships based on Ganqing and Renqing. These dimensions of Guanxi have been found to amplify the positive impact of adaptability on work engagement. Educators can build these relationships through mentorship programs, team-building exercises, or even informal social gatherings, all of which could serve as platforms for meaningful interactions.

Institutions bear the responsibility of creating an environment conducive to both adaptability and work engagement. Given that Ganqing and Renqing were found to be significant mediators, institutions should consider implementing policies that encourage these forms of social networking. This could be achieved by establishing platforms where educators can engage with one another in a non-work-related context, thereby facilitating the development of deeper emotional bonds. Additionally, institutions should consider incorporating adaptability training into their professional development programs, given its direct influence on work engagement. This could be particularly valuable in the context of international educational settings, where educators often have to navigate a complex array of cultural, linguistic, and pedagogical challenges.

In summary, this study offers actionable insights for educators and institutions looking to enhance work engagement in international educational settings. The findings suggest that individual adaptability and certain dimensions of Guanxi—namely Ganqing and Renqing—play pivotal roles in influencing work engagement. As such, both educators and institutions would do well to focus their efforts on these areas.

## Conclusion

This study investigated the relationships among individual adaptability, Guanxi dimensions, and work engagement among educators in international educational institutions in China. Utilizing statistical methodologies, the research affirmed the significance of individual adaptability in enhancing work engagement and elucidated the mediating roles of Ganqing and Renqing, key components of Guanxi. The findings were further contextualized within Confucian values and the current landscape of international education. Despite certain limitations, the study provides meaningful insights for both scholars and practitioners in the field of international education.

## Data availability statement

The raw data supporting the conclusions of this article will be made available by the authors, without undue reservation.

## Ethics statement

The studies involving humans were approved by the Ethics Committee Harbin University. The studies were conducted in accordance with the local legislation and institutional requirements. The participants provided their written informed consent to participate in this study.

## Author contributions

SL: Writing – original draft, Writing – review & editing. SY: Writing – original draft, Writing – review & editing.
